# Career perspective: Ralph F. Goldman—military ergonomics

**DOI:** 10.1186/2046-7648-2-35

**Published:** 2013-12-06

**Authors:** Ralph F Goldman

**Affiliations:** 1 Comfort Technology, 7 West Trevor Hill, Plymouth, MA 02360, USA

**Keywords:** Nutrition and body composition, Military operations, Biomedical effects of clothing, Heated, sweating, walking manikins, Effects of heat, cold, and loads

## Abstract

Military Ergonomics is a name I made up when the Commander at the US Army Research Institute of Environmental Medicine (USARIEM) told me '*The Surgeon General wants to give you a Research Division of your own.’* I demurred, saying '*That would make me an Administrator, and I prefer research,*’ but the C.O. (who was a friend) insisted, saying that what I wanted had no impact on what the General wanted and I had to become the Director of either the Heat, Cold, Work, or Altitude Divisions. Thinking fast, I said '*I want the “Military Ergonomics Division”* ’, and when he asked '*What's that?’* I said '*That's good- it means I can continue my studies on the effects of heat, cold, terrain, load carried, clothing, food, & water intake on troops.*’

## 

My father was born in a 'shtetl’ near Gorodno (Figure 1) to a family of 'gelerntes’ , i.e., sons who began lifelong study at age 4 to be trained as husbands for daughters of wealthy families and to 'keep the brains in the family.’ But, in that era being Jewish, at age 7 he would have been inducted to the Russian Army as a cavalry equerry for 17 years. So at age 5, he and his brother (age 3), pregnant mother, and father moved to America where his mother was found to have tuberculosis (TB), and his mother and father were sent back to Europe; the two boys were left with an aunt who lived in Boston. My grandmother died 2 years later from TB, after giving birth to her fourth son, and my grandfather returned to Boston and raised his four sons as gelerntes. My father became an MD, had an office as an OBGYN in the Italian 'North End’ of Boston and in our home in Roxbury, earned an MPH at Harvard School of Public Health, became Deputy Commissioner of Public Health for Boston and a lecturer at Tufts University Medical School, and died of MI at age 47, leaving my mother at 41 with three sons.

In keeping with tradition, I learned to read before I went to kindergarten. I was sent to Boston Latin School in the seventh grade, where I had 3 1/2 h of homework every night; had two piano lessons a week, for which I had to practice 2 1/2 h a day (and had to perform before the entire school every Saturday, along with lessons in Music Appreciation, Theory, and Toy Orchestra); and had two Hebrew lessons from a tutor each week, which required another hour a day of study. All my homework had to be laid out on the dining room table before I went to sleep each night for my father to check. The first night it was not, he woke me and, when I attempted to explain that I had two back to back study periods the next morning and planned to do it then, dragged me out of bed and slapped me—saying 'You will do it now’; I did it, with tears streaming down my face. On the other hand, I knew he loved me; he gave me horseback riding lessons on Saturdays, took me on call with him, and occasionally left me for half a day with one of his Italian patients in the North End, where I learned to speak Italian and cook Italian food. I learned to play contract bridge sitting on his lap on Sundays and became an avid reader. He built a 4 × 8 'sand table’ for me, bought me lots of tin soldiers, which I would use to stage various historic battles, and took me out to a ship which he was checking for quarantine one Saturday night.

I was in the tenth grade at BLS when he died; I ran wild for about 6 months, switched from classic to jazz piano, and, when my mother moved us to Brookline, had to repeat the tenth grade at Brookline High. This hampered my being accepted to college in 1946 since I was in competition with all the GIs returning from World War Two (WW II). But one of my uncles—who had contracted TB from his mother—had moved to Denver to enter a sanitarium, where he met a young nurse who told him she would not let him die and married him. He lived to be 82, went to work, and (in 'gelernte tradition’) built a very large liquor company for its owner—who then became a major donor to the University of Denver. My uncle's wife and children had visited us, and his wife called my mother to suggest that I come to the University of Denver (DU). I went by train and, getting off in Denver, saw a man walking away from me; from his walk, I knew he was my uncle. The next day, he took me to meet the Chancellor of DU, who told me I had been accepted (without application).

Given my background, DU was no challenge; the first year, I joined a fraternity (Phi Sigma Delta—in which my aunt and cousins were active) and often went to class only Wednesdays, leaving to ski, hunt, or fish in the mountains, but returning by Tuesday. The second year, taking Physiology (with Dr. Fred Damour) and Biochemistry (with Dr. Frank Blood), I became a scholar and moved to the fraternity house. I also joined the Colorado Air National Guard, making me Corporal after they taught me to drive. I worked my way through the 3 years it took me to acquire enough points to graduate, playing cards (after the first trimester, when I sent home US$800 after having paid my expenses for the year, I refused to play for money with my fraternity brothers), guiding hunting and fishing parties, playing piano in a nightclub, teaching dancing for Arthur Murray, and breaking winter range horses. My fraternity brothers entered me in the Amateur Bronco Busting competition at the Cheyenne, WY, stampede—where I stayed on for a very long 10 s—until the whistle blew and two pickup riders came alongside and lifted me off the broncho, and I won a bronze belt buckle, which I still prize. I graduated in December 1949 with a BA in science after 3 1/3 years and drove a friend's car to Boston, where I worked at the post office for the Christmas rush, worked on the 1950 census, drove a taxi, and occasionally played a piano gig.

In September, I enrolled for an MA in Biology at Boston University (BU). After getting 100s in my exams in Comparative Anatomy (with Dr. Don Patt) and in Experimental Physiology (with Dr. L.C. Wyman), the latter gave me a job identifying and pinning the University's insect collections and then had me teach Anatomy and Histology for graduate nurses. Dr. Wyman arranged for me to attend the American Physiological Society in Atlantic City as a demonstrator at the Harvard Apparatus Company Booth and subsequently got me accepted for the PhD program in Endocrinology at BU, then with a National Institutes of Health Research Fellow appointment, and then a Postdoctoral appointment with the Atomic Energy Commission studying the effects of radiation on adrenal gland function. I remained his friend for the rest of his life, helped him move into a retirement home after his wife died, and took him to dinner monthly as long as he lived.

Unfortunately, after 2 years working with animals for the AEC, I became allergic to the animals. I was used to breathing without an impediment, so I decided to offer my expertise in endocrinology, and the responses to heat and cold stress, to the US Army Quartermaster Corps which, at the start of WW II—when the USA was losing over 17 times as many troops to cold injuries than Japanese bullets on Kiska and Attu in the Aleutian Campaigns—had commandeered the only climate chamber in the USA which could operate at -60°F, at the American Woolen Mills in Lawrence, MA, and was moving from Lawrence to Natick, MA. I made an appointment, went to Lawrence, and met with the staff, who said I was overqualified for any position they had opened at the time, so the highest position they could offer me was at the GS-9 level. I accepted it on the spot, but they were right since some 25 years later I was heading my Military Ergonomics Division at the SES 5 level (equivalent to a three-star General), having won a number of awards and medals, including the highest award the Army could give to a civilian.

Along the way, I built the first underwater weight apparatus in the USA and used it to study body composition in troops on special rations, in young girls at an overweight girls camp that Harvard ran on Cape Cod, and in the study by Ethan Simms (a world renowned expert on diabetes) and colleagues on prisoners at the Vermont State prison who were being evaluated for body composition changes during the daily maximum calories they could ingest and during subsequent weight loss on return to a restricted diet. My earliest publications (*cf.*[1-24]) were on body composition, and I was privileged to also work with E.R. Buskirk who trained with Ancel Keys (designer of the original K-rations) and Pat Iampietro who trained with E.F. Adolph (who authored the classic *Man in the Desert*).

I arranged with Harvard Apparatus Company to build the first electronically controlled work cycle and used it to study the troop's maximum ability to work—how it could be changed by training and de-training—and working with an international group of scientists, I served as Editor for the first issue of the American College of Sports Medicine and became a Fellow of the ACSM. But perhaps my most significant accomplishment was the development of a heated, sweating—and eventually walking—'Cuman’ (a life-sized Copper Man, building on the original heated manikin built by my friend Woodie Belding to study sleeping bags for the Quartermaster Lab originally located in Lawrence, where he had been one of the original Directors of the Climatic Research Lab. Originally, some nine of these had been built and used to study the thermal insulation of various military and civilian clothing items and their 'CLO’ value (a unit developed by my good friend and mentor A. Pharo Gagge), akin to the R-value of insulation used in heating, ventilation, and air-conditioning (HVAC) applications. By the time I started at Natick, most of these had been destroyed or put in storage, but when Alan Woodcock (who headed the Biophysics group at Natick when I joined it) showed me a wet cylinder he had developed to study the sweat permeability of clothing materials, using his 'permeability index’ , Im—a dimensionless index characterizing the ability of moisture to evaporate through the material—where #0 represented a totally impermeable material and #1 indicated a totally unimpeded evaporative cooling from the underlying heated surface, I got quite excited and said 'that's the missing link and I can get a copper man sweating.’ Alan insisted I could not, and even if I could, what could I do with the information. I said I was sure I could and then would be able to predict the total dry and evaporative heat transfer through any uniform. I wish I had patented the idea since there are now several thousand manikins in the world (some with price tags in the hundreds of thousands of dollars) and there have been a number of international meetings on their use.

A. Pharo Gagge, who I had met after a presentation I made at a Physiological Society in Atlantic City, invited me to present a lecture on the heated, sweating manikin at the J.B. Pierce Foundation at Yale University. After I made the presentation, he asked me to join the American Society of Heating, Refrigerating and Air Conditioning Engineers (ASHRAE), and when I explained that I had never run a comfortable study, he replied 'But you understand human heat transfer better than any HVAC engineer, and we need you to teach them.’ So I joined ASHRAE, became a Distinguished Fellow and won a medal from the Society, became a member of the Board of Directors, and, in response to a request by the President, investigated whether the Society should be involved in indoor air quality (IAQ); I gathered some experts I knew and, after studying the literature, recommended that ASHRAE not only should be involved but should set up a special committee to do so, which was funded to cover the costs for invited experts who would not normally attend ASHRAE meetings. I wound up chairing the committee, running an annual meeting on IAQ, and being a reviewing editor of a new journal on IAQ we established.

I am one of the founding members of the International Conference on Environmental Ergonomics and—albeit 85—have just returned from a 3-week trip to lecture at several international meetings in New Zealand. I was a charter member of the BioPhysics Society, a Fellow of other professional societies, and a Visiting Faculty Member/Professor at MIT, BU, NCSU, URI, Harvard School of Public Health, and Jinan University in China. I ran key studies on loan to South Africa, Australia, and NATO; helped get the new Sherman tank and the US aircraft carrier pilot ready rooms air-conditioned; changed the National Policy on Operations in CBR Protective Clothing, for which I received a number of medals; and enjoyed lecturing all over the world (Figure 2).

**Figure 1:**
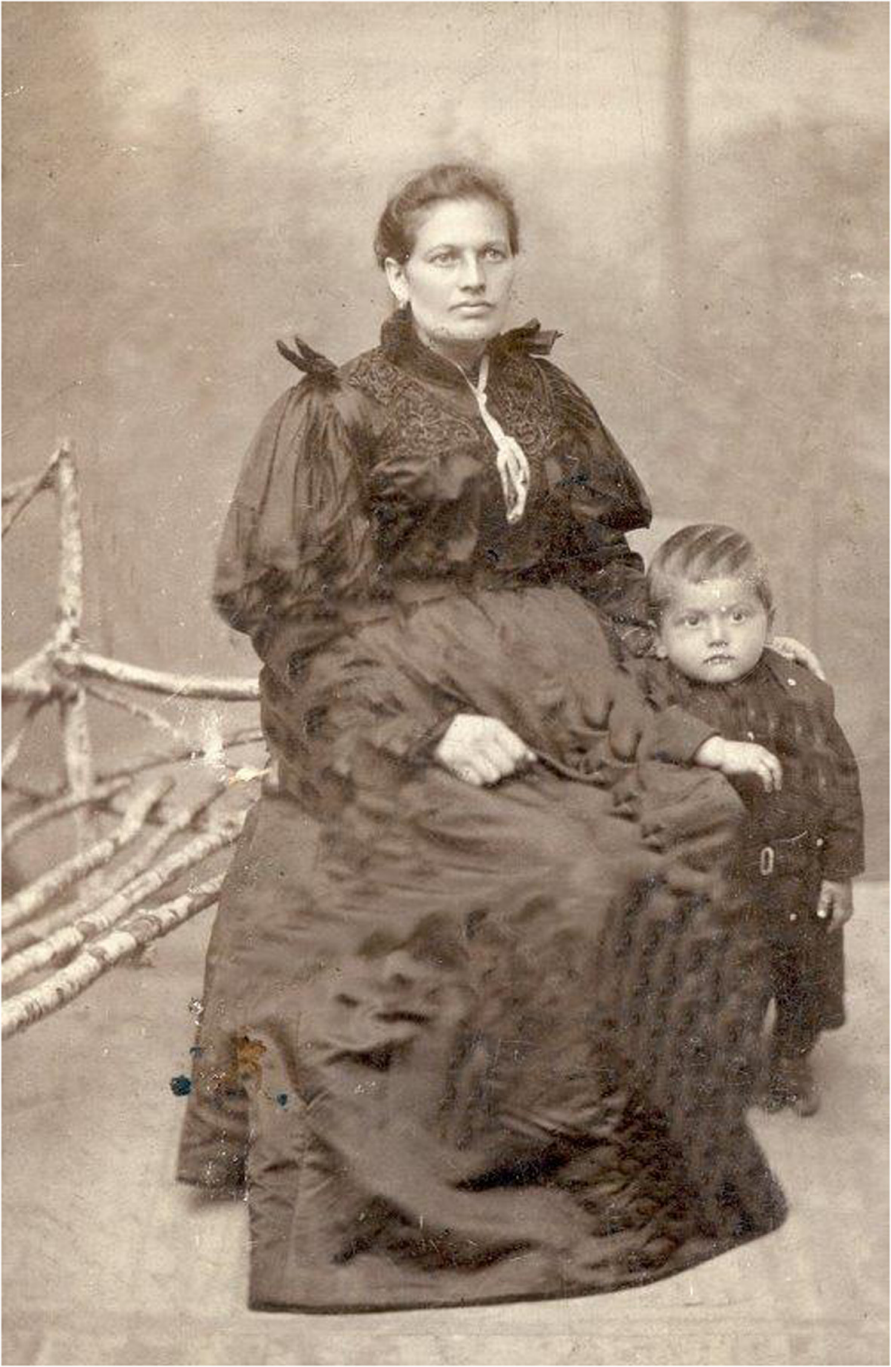
Frances and Harry Goldman, approximately 1898.

**Figure 2:**
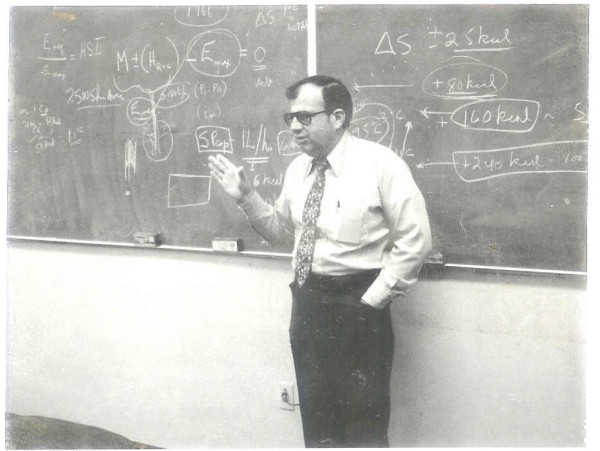
Prof. Ralph Goldman teaching at New York State University, 1962.

## Abbreviations

ASHRAE: American Society of Heating, Refrigerating and Air Conditioning Engineers; BU: Boston University; CLO: Unit of clothing insulation; IAQ: Indoor air quality; Im: permeability index; MI: Myocardial infarction; MIT: Massachusetts Institute of Technology; NCSU: North Carolina State University; OBGYN: obstetrician and gynecologist; TB: tuberculosis; URI: University of Rhode Island; USARIEM: US Army Research Institute of Environmental Medicine; WW II: World War Two.

## Competing interests

The author declares that he has no competing interests.
